# Renal‐Limited Thrombotic Microangiopathy due to Anti‐VEGF/TKI Immunotherapy for Metastatic Renal Cell Carcinoma Presenting as Nephrotic Syndrome: A Case Report and Literature Review

**DOI:** 10.1002/ccr3.72202

**Published:** 2026-03-20

**Authors:** Sundus Sardar, Ahmad Matarneh, Omar K. Salameh, Abdel‐Rauof M. Akkari, Monika Joshi, Erik Washburn, Nasrollah Ghahramani, Naman Trivedi

**Affiliations:** ^1^ Penn State Milton S Hershey Medical Center Hershey Pennsylvania USA; ^2^ Pennsylvania State University College of Medicine Hershey Pennsylvania USA

**Keywords:** hypertension, nephrotic syndrome, proteinuria, thrombotic microangiopathy, vascular endothelial growth factor inhibitor

## Abstract

Vascular endothelial growth factor (VEGF) inhibition may result in proteinuria, worsening hypertension, chronic kidney injury, or glomerular disease. Recently, systemic VEGF inhibition has been reported to cause nephrotic disorders and thrombotic microangiopathy (TMA). We present a unique case of renal‐limited TMA presenting as nephrotic syndrome in a patient on anti‐VEGF/TKI immunotherapy and immune checkpoint inhibitors for metastatic renal cell carcinoma.

## Key Clinical Message

Anti‐VEGF and TKI therapies can cause nephrotic syndrome and renal‐limited TMA; monitor blood pressure, proteinuria, and kidney function closely. Accurate differentiation of renal‐limited TMA from minimal change disease or focal segmental glomerulosclerosis guides management. Prompt cessation of the implicated therapy and nephrology referral improve outcomes and allow potential immunotherapy rechallenge.

## 1 Introduction

Anti‐VEGF agents represent an important milestone in oncology by targeting VEGF, a crucial mediator of angiogenesis and tumor growth. By inhibiting the VEGF pathway, these therapies effectively impede the formation of new blood vessels, which are essential for tumor growth and metastasis. While they have markedly improved outcomes for many individuals with cancer, anti‐VEGF treatments are associated with a range of side effects, including renal complications, which, although rare, can occur. These complications can manifest as mild proteinuria, hypertension, or escalate to severe conditions such as nephrotic syndrome and thrombotic microangiopathy (TMA) [1].

TMA is a pathological condition characterized by a triad of thrombocytopenia, microangiopathic hemolytic anemia, and organ dysfunction, with the kidneys often being significantly affected. Although the incidence of TMA related to anti‐VEGF therapy is not well established, it is a serious condition that necessitates prompt recognition and management to avert irreversible kidney damage and failure. This case report describes a patient who developed renal‐limited TMA presenting as nephrotic syndrome while undergoing anti‐VEGF therapy for metastatic renal cell carcinoma, underscoring the critical importance of early recognition and intervention to mitigate renal adverse outcomes while balancing cancer treatment.

## 2 Case History/Examination

A 73‐year‐old man with a diagnosis of metastatic renal cell carcinoma (RCC) undergoing treatment with a combination of axitinib, a selective TKI targeting VEGF receptors, and pembrolizumab, an immune check point Programmed Cell Death‐1 inhibitor (anti‐PD‐1i). He presented to the clinic with significant generalized edema and a weight gain of approximately 30 pounds over a short period. His clinical examination revealed hypertension, and marked bilateral lower extremity swelling.

Of note, he was initially treated with combination immunotherapy and tyrosine kinase inhibitor, receiving nivolumab every 4 weeks for approximately 18.5 months. Concurrently, cabozantinib 20 mg daily was administered on an intermittent schedule due to multiple clinical interruptions, including toe infection and subsequently toe amputation, resulting in approximately 15 months of total active treatment. Following disease progression and inability to tolerate higher doses of cabozantinib, he was transitioned to axitinib at 5 mg twice daily for 18 days, which was subsequently reduced to 3 mg twice daily due to concerns for uptrending serum creatinine for 52 days.

Upon current presentation, initial laboratory workup demonstrated heavy proteinuria, with a urine protein‐to‐creatinine ratio of 14 g/g, alongside hypoalbuminemia, as evidenced by a serum albumin level of 2.6 g/dL, raising concern for nephrotic syndrome. Other relevant lab results included normal serum creatinine at 0.9 mg/dL with eGFR of 80, and a complete blood count that showed no signs of hemolysis or thrombocytopenia.

## 3 Differential Diagnoses

In this 73‐year‐old man with metastatic RCC treated with sequential immune checkpoint inhibitors (ICIs) and VEGF‐targeted tyrosine kinase inhibitors (TKIs), the acute onset of generalized edema, nephrotic‐range proteinuria, and new or worsening hypertension prompted consideration of several potential etiologies. Given his complex oncologic regimen, the leading diagnostic category was drug‐induced glomerular and endothelial injury.

Both VEGF‐pathway inhibition and immune checkpoint blockade are recognized causes of kidney injury through distinct mechanisms. Anti‐VEGF and TKI agents such as axitinib and cabozantinib can cause renal‐limited thrombotic microangiopathy (TMA) or podocytopathy (minimal change disease or focal segmental glomerulosclerosis). VEGF signaling is critical for maintaining glomerular endothelial integrity and podocyte–endothelial crosstalk; its inhibition results in endothelial swelling, loss of fenestrations, and microvascular ischemia. Clinically, this manifests as hypertension, often early in the course of therapy, due to reduced nitric oxide production and capillary rarefaction. The development of hypertension concurrent with nephrotic‐range proteinuria in this patient therefore strongly supported a VEGF‐pathway–mediated endothelial injury, with renal‐limited TMA as the most plausible mechanism.

By contrast, immune checkpoint inhibitor–related nephrotoxicity, as from pembrolizumab or prior nivolumab exposure, typically produces immune‐mediated tubulointerstitial nephritis or glomerular lesions such as minimal change disease or membranous nephropathy. These entities may present with proteinuria but are less frequently accompanied by severe hypertension, and the long interval since prior ICI use made this explanation less likely.

Paraneoplastic glomerulopathy was also considered, as RCC can rarely induce secondary membranous nephropathy or AA amyloidosis through tumor‐related cytokine or antigen release. However, the absence of systemic inflammatory features and the temporal association of symptoms with axitinib initiation argued against a paraneoplastic process. Chronic conditions such as hypertensive nephrosclerosis or diabetic nephropathy could theoretically contribute to baseline proteinuria, but the abrupt 30‐pound weight gain, nephrotic‐range proteinuria, and preserved renal function were inconsistent with a chronic course.

Finally, primary nephrotic syndromes unrelated to therapy (such as idiopathic membranous nephropathy, minimal change disease, or FSGS) warranted exclusion by serologic evaluation for autoimmune and viral etiologies, which were unremarkable in this case.

Taken together, the co‐occurrence of new‐onset hypertension, heavy proteinuria, and preserved renal function following VEGF/TKI exposure—in the absence of hemolysis or thrombocytopenia—was most consistent with renal‐limited thrombotic microangiopathy secondary to anti‐VEGF/TKI therapy. Other causes, including ICI‐related glomerular disease or paraneoplastic mechanisms, were considered less likely.

## 4 Conclusion and Results (Outcome and Followup)

**FIGURE 1:**
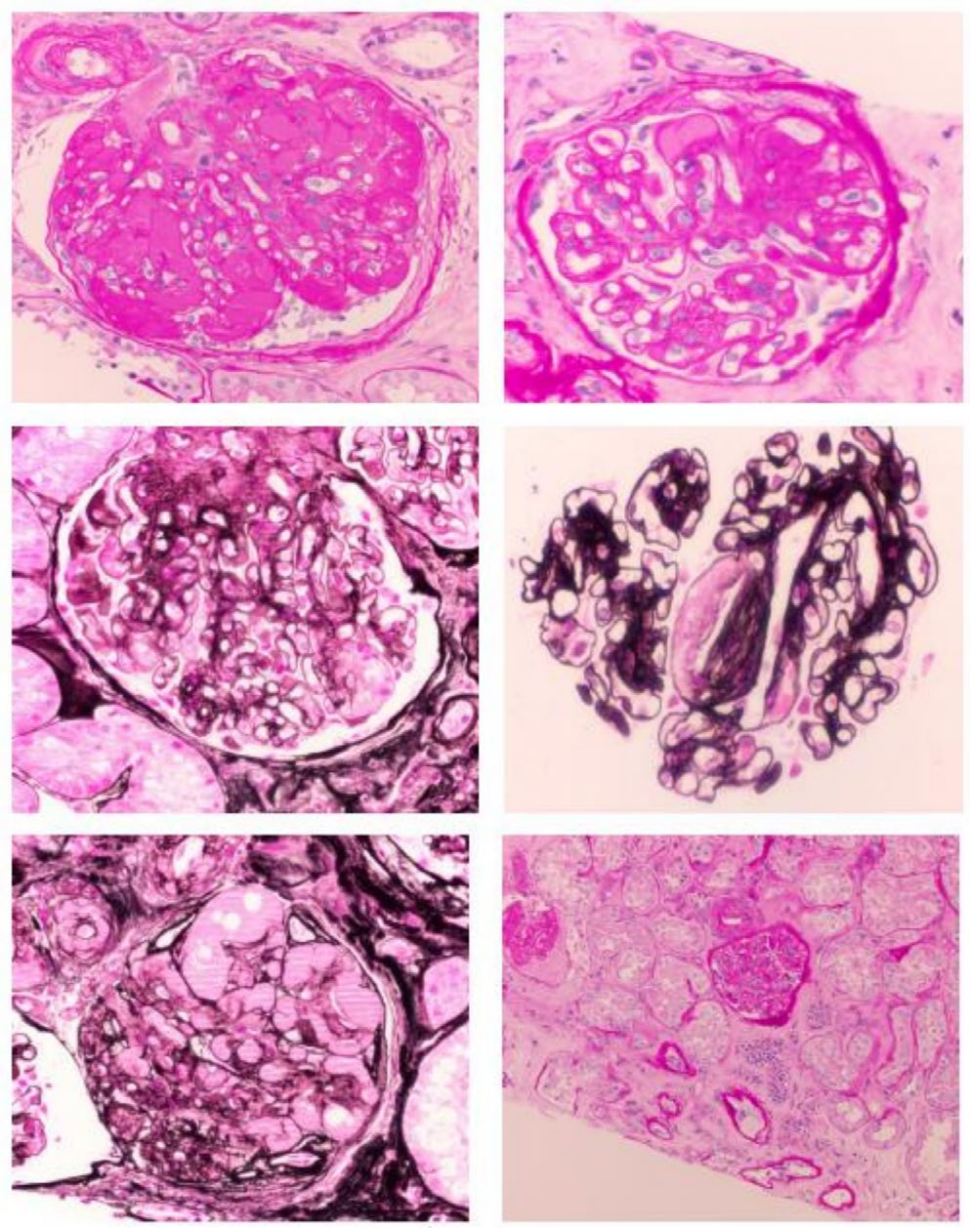
Kidney biopsy showed thrombotic microangiopathy (TMA) with endothelial swelling, mesangiolysis, basement membrane duplication, arteriolar hyalinosis, and moderate interstitial fibrosis and tubular atrophy. Findings are consistent with chemotherapy‐associated (anti‐VEGF‐induced) TMA and concurrent diabetic nephropathy.

**FIGURE 2:**
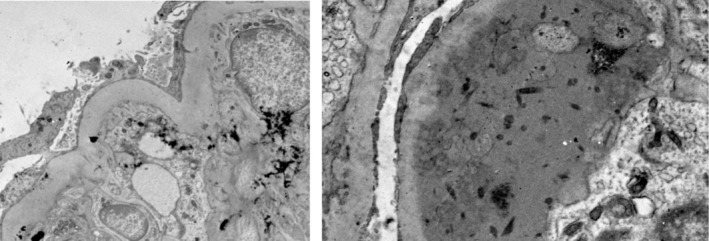
Electron microscopy showed the endothelial cells focally detached from the glomerular basement membrane and the subendothelium is expanded by election lucent material (arrows). The capillary lumen is compressed by an expanded subendothelium with fibrin tactoids (arrows) and entrapped cell debris.

Given the clinical suspicion of nephrotic syndrome, a renal biopsy was performed. Histological pathology (Figure 1, Figure 2) confirmed renal‐limited thrombotic microangiopathy, most likely attributable to the anti‐VEGF/TKI therapy. Notably, electron microscopy revealed no evidence of immune complex deposition, which ruled out immune complex‐mediated disease. However, extensive foot process effacement was observed, correlating with the significant proteinuria.

In light of these findings, the decision was made to discontinue the anti‐VEGF/TKI therapy. The patient was subsequently managed with losartan and dapagliflozin to mitigate proteinuria and manage hypertension. Upon follow‐up, his urine protein‐to‐creatinine ratio improved to 7 g/g, and he experienced a notable reduction in edema, indicating a positive response to the adjusted therapeutic regimen.

**FIGURE 3:**
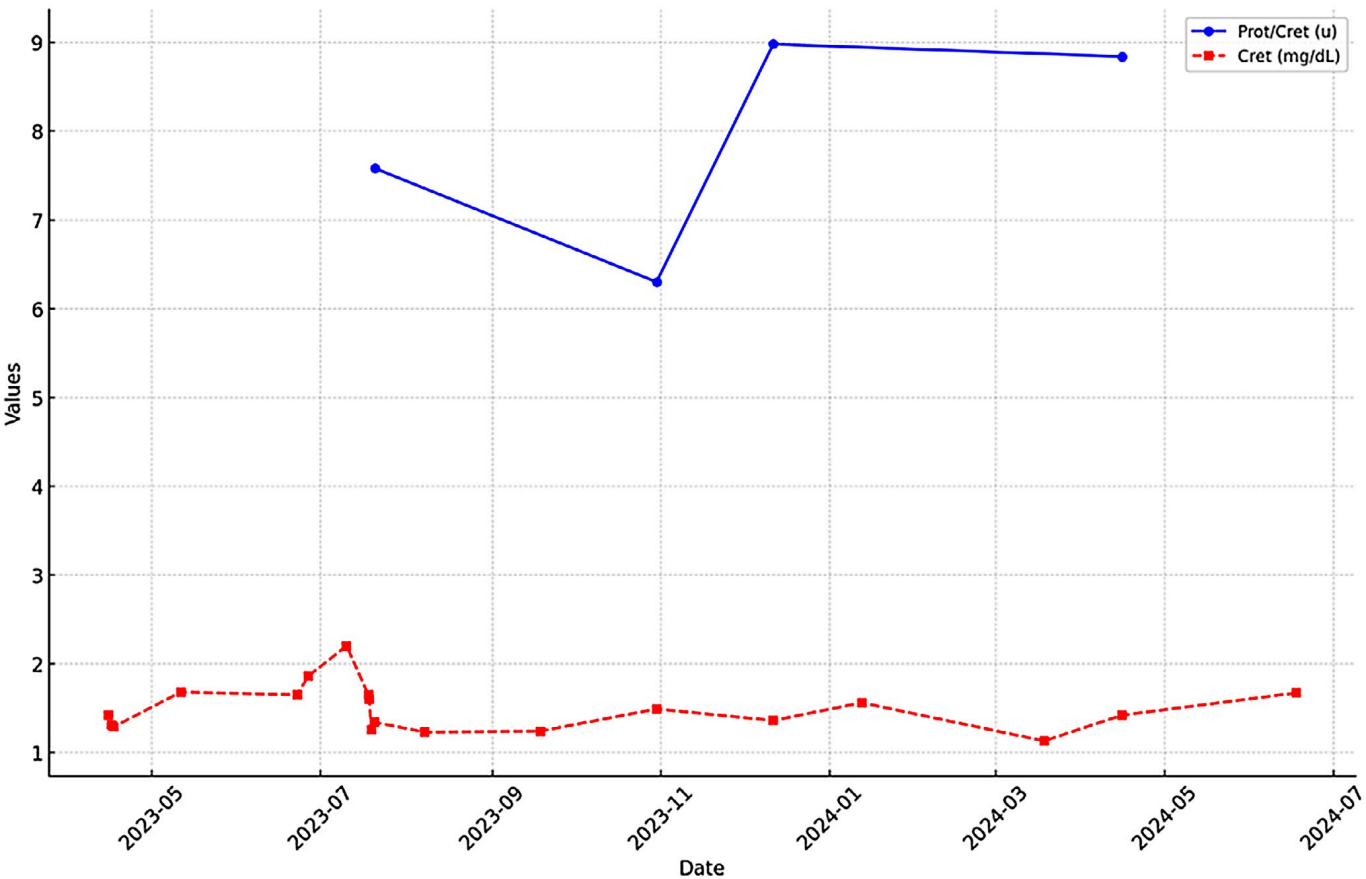
Longitudinal trends in serum creatinine and urine protein‐creatinine ratio (UPCR).

**FIGURE 4:**
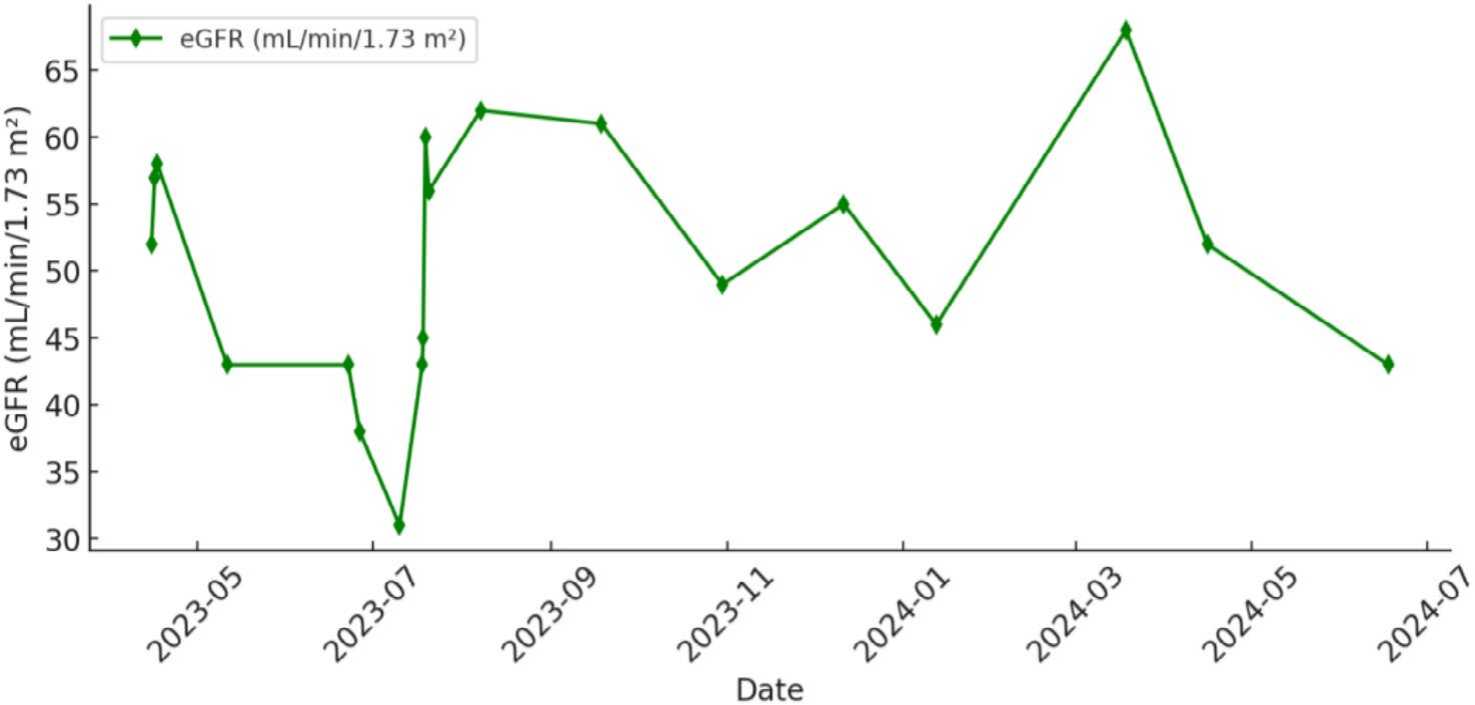
Trends in estimated glomerular filtration rate (eGFR) over the clinical course.

Axitinib was discontinued after approximately 2.5 months due to worsening renal function. Pembrolizumab was administered concurrently during this phase of treatment. He was then transitioned to levatinib 10 mg daily and pembrolizumab; creatinine remained stable around 1.3–1.5 mg/dL. Longitudinal trends in serum creatinine, urine protein creatinine ratio and eGFR over clinical course are illustrated in Figures 3 and 4. Subsequent PET imaging showed further progression of disease with bone metastases, hence transitioned therapy to sunitinib with side effects due to which he was switched to tivozanib. Given the overall poor quality of life, the patient elected to transition to hospice care.

## 5 Discussion

Antivascular endothelial growth factor (VEGF) agents have revolutionized cancer treatment by effectively targeting angiogenesis. However, their use is accompanied by potential side effects, including renal complications. The mechanism by which anti‐VEGF agents cause renal damage is thought to involve disruption of glomerular endothelial cells, leading to endothelial injury and the development of thrombotic microangiopathy (TMA) [1, 2]. Our case report highlights a rare and clinically significant instance of renal‐limited TMA secondary to anti‐VEGF and tyrosine kinase inhibitor (TKI) immunotherapy in a patient with metastatic renal cell carcinoma (mRCC). TMA is characterized by microvascular damage, resulting in hemolytic anemia, thrombocytopenia, and organ dysfunction [3]. While systemic presentations of TMA are well documented, isolated renal manifestations, particularly presenting as nephrotic syndrome, are less frequently encountered in the context of anti‐VEGF/TKI therapy [4].

The pathophysiology underlying TMA in this setting appears to be multifactorial. Anti‐VEGF agents and TKIs may induce endothelial cell dysfunction and promote a pro‐thrombotic state, leading to vascular damage and microangiopathic changes [5]. The increased vascular permeability and resultant proteinuria in our patient can be attributed to the direct effects of these agents on the glomerular endothelium, resulting in nephron damage and nephrotic syndrome [6].

Demographics for renal‐limited TMA associated with anti‐VEGF and TKI therapies in metastatic RCC include a mean patient age of 59.8 years (range 20–85) and a slight female predominance (58%) [4]. Patients often present with proteinuria, hypertension, and renal insufficiency, with significant proteinuria reported in 31% of cases. Renal biopsies are typically performed about 6.87 months after therapy initiation. Histologically, 73% exhibit renal TMA, while others may show minimal change disease or collapsing focal segmental glomerulosclerosis [4]. These findings emphasize the importance of monitoring renal function and blood pressure in patients receiving anti‐VEGF/TKI therapy due to potential renal adverse effects.

Known risk factors for renal‐limited TMA in this context include preexisting hypertension, chronic kidney disease (CKD), older age (≥ 60 years), and the specific type of VEGF inhibitor used [7]. Significant proteinuria prior to or during treatment can indicate underlying endothelial damage, while concurrent nephrotoxic agents further increase risk [8].

This case underscores the need for heightened awareness among clinicians regarding the renal side effects of anti‐VEGF/TKI therapies, especially in patients presenting with unexplained nephrotic syndrome. Early recognition and prompt intervention are crucial to preventing irreversible kidney damage. The differential diagnosis of nephrotic syndrome in patients on such therapies should include TMA, characterized by microvascular thrombosis and endothelial injury, which can lead to severe renal impairment, particularly when accompanied by a clinical picture of hemolysis or thrombocytopenia [9, 10].

In reviewing the literature, we found limited cases of TMA associated with anti‐VEGF/TKI therapy. Most documented instances involve systemic TMA, with renal involvement as part of a broader clinical syndrome [11]. Summary of published case reports describing clinical presentation, biopsy findings, and outcomes in patients who developed renal complications during therapy with VEGF‐targeted agents or tyrosine kinase inhibitors (TKIs) is documented in Table 1. Our case stands out due to the isolated renal manifestation, suggesting that clinicians should maintain a high index of suspicion for TMA when evaluating nephrotic syndrome in this population [29].

**TABLE 1:** Reported cases of VEGF inhibitor–associated renal injury and thrombotic microangiopathy (TMA).

Author	Year	Country	VEGFi type	Dose	Age, gender	Underlying diagnosis	VEGFi/TKI duration	Presentation	Renal biopsy findings	Management/Outcome
Müller‐Deile et al.	2010	Germany	Sunitinib	50 mg daily (4 weeks on, 2 weeks off)	61, Male	Metastatic renal cell carcinoma	9 months	Acute renal failure, proteinuria	Focal segmental glomerulosclerosis (FSGS), endothelial damage, interstitial nephritis	Required dialysis; patient died 14 months after tumor diagnosis [12]
Usui et al.	2014	USA	Bevacizumab	Not specified	60, Female	Metastatic breast cancer	6 months	AKI, hypertension, proteinuria	Thrombotic microangiopathy (TMA)	Discontinued bevacizumab; partial renal recovery [13].
Usui et al.	2014	USA	Bevacizumab	Not specified	55, Male	Metastatic colorectal cancer	4 months	AKI, proteinuria	TMA	Discontinued bevacizumab; renal function improved [13].
Usui et al.	2014	USA	Bevacizumab	Not specified	62, Male	Metastatic colorectal cancer	5 months	Hypertension, proteinuria	TMA	Discontinued bevacizumab; partial renal recovery [13].
Usui et al.	2014	USA	Bevacizumab	Not specified	68, Female	Metastatic ovarian cancer	3 months	AKI, proteinuria	TMA	Discontinued bevacizumab; renal function improved [13].
Usui et al.	2014	USA	Sorafenib	Not specified	70, Male	Hepatocellular carcinoma	2 months	AKI, hypertension, proteinuria	TMA	Discontinued sorafenib; partial renal recovery [13].
Peña‐Hernández et al.	2019	USA	Sunitinib	Not specified	60, Male	Renal cell carcinoma	Not specified	Hypertension, proteinuria	Not specified	Discontinuation of sunitinib led to improvement in renal function [14]
Shye et al.	2020	USA	Intravitreal bevacizumab	1.25 mg per eye every 2 months	56, Male	Diabetic retinopathy	Several months	Worsening proteinuria, renal function decline	Diabetic nephropathy, FSGS with collapsing features, acute interstitial nephritis	Discontinuation of bevacizumab; partial renal recovery [15]
Hanna et al.	2020	USA	Intravitreal bevacizumab	1.25 mg per eye every 2 months	56, Male	Diabetic retinopathy	8 months	Worsening creatinine, nonresolving proteinuria	Renal‐limited thrombotic microangiopathy (TMA)	Continued bevacizumab due to severe visual impairment; planned for hemodialysis initiation [16]
Hanna et al.	2020	USA	Intravitreal bevacizumab	1.25 mg per eye every 2 months	43, Female	Diabetic retinopathy	6 months	Worsening hypertension, nonresolving proteinuria	Chronic TMA, secondary FSGS	Not available [16]
Hanna et al.	2020	USA	Intravitreal aflibercept	Not specified	77, Female	Diabetic retinopathy	Few years	Worsening creatinine, proteinuria	Diabetic nephropathy, endothelial and vascular damage	Discontinued aflibercept; reverted to ranibizumab [16]
Phadke et al.	2021	USA	Intravitreal aflibercept	Not specified	74, Male	Age‐related macular degeneration	Not specified	Nephrotic syndrome	Collapsing FSGS, TMA	Discontinuation of aflibercept; partial renal recovery [17]
Yoshimura et al.	2022	Japan	Ramucirumab	Not specified	70s, Male	Gastric cancer	Immediately after administration	Proteinuria, renal dysfunction	Two types of renal lesions simultaneously	Discontinuation of ramucirumab; improvement in renal function [18]
Zhang et al.	2023	China	Fruquintinib	Not specified	73, Male	Metastatic colon cancer	Not specified	Proteinuria, renal dysfunction	Renal‐limited TMA	Discontinuation of fruquintinib; improvement in renal function [19]
Sardar et al.	2013	India	Sunitinib	50 mg daily (4 weeks on, 2 weeks off)	60, Male	Metastatic RCC	22 months	Nephrotic syndrome, renal dysfunction	TMA with diffuse podocyte foot process effacement	Discontinued sunitinib; initiated oral steroids; significant improvement in renal function and proteinuria [20]
Prasoppokakorn et al.	2022	Thailand	Lenvatinib	8 mg/day	67, Female	Hepatocellular carcinoma	2 weeks	Hypertension, bilateral pleural effusion, edema, hypoalbuminemia, hypercholesterolemia, proteinuria	Not performed	Discontinued lenvatinib; symptoms improved within 1 week; proteinuria reduced to subnephrotic range over 6 months [21]
Furuto et al.	2018	Japan	Lenvatinib	10 mg/day	79, Female	Metastatic thyroid cancer	3 months	Hypertension, nephrotic syndrome, acute kidney injury	FSGS with complete and incomplete glomerular hyalinization	Discontinued lenvatinib; partial recovery over 15 months [22]
Yin et al.	2023	China	Lenvatinib	Not specified	35, Female	Papillary thyroid carcinoma	Concurrent with diagnosis	Nephrotic syndrome	Minimal change disease	Treated with thyroidectomy and prednisone; achieved complete remission [23]
Yin et al.	2023	China	Lenvatinib	Not specified	50, Male	Papillary thyroid carcinoma	3 years postdiagnosis	Nephrotic syndrome	Minimal change disease	Treated with tacrolimus and rituximab; achieved complete remission [23]
Yin et al.	2023	China	Lenvatinib	Not specified	50, Male	Papillary thyroid carcinoma	3 years postdiagnosis	Nephrotic syndrome	Minimal change disease	Treated with tacrolimus and rituximab; achieved complete remission [23]
Zhu et al.	2023	China	Surufatinib	Not specified	43, Female	Adenoid cystic carcinoma	Not specified	Nephrotic syndrome	TMA with glomerular endothelial swelling, subendothelial widening, mesangiolysis, double contours	Discontinued surufatinib; managed with antihypertensives; partial renal recovery [24]
Bhat et al.	2024	UK	Sunitinib	Not specified	Early 70s, Male	Metastatic clear cell RCC	8 years	Nephrotic syndrome, renal dysfunction	Chronic TMA	Discontinued sunitinib; improved renal function and proteinuria [25]
Zhu et al.	2023	China	Regorafenib	Not specified	62, Male	Metastatic colon cancer	Not specified	Proteinuria	TMA	Discontinued regorafenib; partial renal recovery [26]
Sardar et al.	2023	USA	Sunitinib	Not specified	Not specified	Metastatic RCC	22 months	Nephrotic syndrome	Renal‐limited TMA	Discontinued sunitinib; renal function improved [27]
Prasoppokakorn et al.	2022	Thailand	Lenvatinib	8 mg/day	67, Female	Hepatocellular carcinoma	2 weeks	Hypertension, bilateral pleural effusion, edema, hypoalbuminemia, hypercholesterolemia, proteinuria	Not performed	Discontinued lenvatinib; symptoms improved within 1 week; proteinuria reduced to subnephrotic range over 6 months [21]
Furuto et al.	2018	Japan	Lenvatinib	10 mg/day	79, Female	Metastatic thyroid cancer	3 months	Hypertension, nephrotic syndrome, acute kidney injury	FSGS with complete and incomplete glomerular hyalinization	Discontinued lenvatinib; partial recovery over 15 months [22]
Delsante et al.	2022	USA	Lenvatinib	18–24 mg/day (varied)	4 patients (3F, 1 M); Age range: 53–69	Thyroid carcinoma	Weeks to months	New‐onset hypertension, proteinuria, AKI in some	TMA with endothelial swelling, mesangiolysis, GBM duplication	Discontinuation of VEGFi led to improvement in renal function and proteinuria in most cases [28]

One study by Keir et al. [30] highlights the pivotal role of VEGF in maintaining local immune quiescence by regulating complement inhibitory proteins such as complement factor H (CFH) and CD59. In both the eye and kidney, VEGF signaling was shown to preserve endothelial and podocyte expression of these inhibitors, thereby protecting against complement‐mediated injury. In our case, the development of thrombotic microangiopathy (TMA) following anti‐VEGF therapy may reflect not only endothelial toxicity but also a loss of VEGF‐mediated regulation of complement control, potentially predisposing to unchecked complement activation and microvascular injury. This dual mechanism may explain the renal susceptibility observed in our patient and underscores the broader immunoregulatory consequences of VEGF inhibition beyond angiogenesis.

Management strategies for drug‐induced TMA are still evolving, but supportive care, including the use of corticosteroids and potential cessation of the offending agent, has shown promise in some cases [31]. Discontinuation of the offending agent is often necessary in severe cases, as continued exposure to the anti‐VEGF therapy can exacerbate renal damage. Supportive care, including blood pressure control and management of edema, is essential. In some cases, plasmapheresis or rituximab may be considered, particularly in systemic TMA, although their efficacy in renal‐limited TMA is less clear [32].

Cessation of anti‐VEGF/TKI therapy can result in significant improvement in proteinuria and stabilization of renal function, with the potential for rechallenge using an alternative agent in select cases. In our patient, discontinuation of the initial VEGF/TKI led to a marked reduction in proteinuria and improved renal function, consistent with prior reports demonstrating reversibility of renal‐limited thrombotic microangiopathy (TMA) upon drug withdrawal [1, 33]. Importantly, switching to a different anti‐VEGF agent did not result in recurrence of nephrotic syndrome or TMA, suggesting that differential off‐target effects may exist among agents in this class. This finding emphasizes the importance of individualized management strategies, including both cessation and cautious reintroduction when clinically indicated. The role of complement activation in VEGF/TKI‐induced renal‐limited TMA remains incompletely understood, and further studies are warranted to elucidate its contribution. Prompt referral to nephrology is critical when renal complications are suspected, ensuring timely evaluation and initiation of renoprotective strategies. In addition to drug withdrawal, optimization of antiproteinuric therapy—including maximally tolerated ACE inhibitors or ARBs and consideration of SGLT2 inhibitors—may offer added benefit, as supported by post hoc analyses of the DAPA‐CKD trial showing proteinuria reduction in both diabetic and nondiabetic CKD, including glomerular diseases such as IgA nephropathy [34].

Our case suggests that renal involvement in patients on anti‐VEGF/TKI immunotherapy may manifest as nephrotic‐range proteinuria and clinically nephrotic syndrome with renal‐limited histological TMA. In such cases, other differentials may include minimal change disease or collapsing focal segmental glomerulosclerosis (FSGS) [4, 35]. Cessation of anti‐VEGF/TKI therapy results in significant improvement in proteinuria with possibility of rechallenge with alternative anti‐VEGF/TKI in the future. In case of concerns for renal sequelae with anti‐VEGF/TKI involvement, prompt referral to nephrology for further evaluation is necessitated for appropriate and timely management. Our patient demonstrated improvement in renal function and reduction in proteinuria following the discontinuation of the anti‐VEGF/TKI therapy, aligning with previous reports indicating that renal outcomes can improve upon drug withdrawal [36, 37].

In conclusion, this case highlights the potential for anti‐VEGF/TKI therapies to induce renal‐limited thrombotic microangiopathy (TMA), which may manifest as nephrotic syndrome. Clinicians should maintain a high index of suspicion for TMA in patients on VEGF/TKI agents who develop proteinuria or nephrotic‐range proteinuria. Early recognition and management are essential to prevent irreversible kidney damage. Importantly, optimizing antiproteinuric therapy with the maximum tolerated doses of ACE inhibitors or ARBs, along with the addition of an SGLT2 inhibitor, may improve proteinuria. This is supported by post hoc analyses of the DAPA‐CKD trial, which demonstrated proteinuria reduction in both diabetic and nondiabetic CKD, including glomerular diseases such as IgA nephropathy. In our case, resolution of nephrotic syndrome was achieved through a combined approach: intensification of antiproteinuric therapy and switching to a different class of VEGF‐targeted therapy. These strategies may represent important considerations in the management of VEGF/TKI‐induced glomerular injury and warrant further investigation.

In conclusion, recognition of hypertension as an early and mechanistically linked biomarker of VEGF‐pathway toxicity has practical value in the care of oncology patients receiving VEGF inhibitors or VEGFR‐TKIs. Blood pressure elevation can potentially precede overt proteinuria or renal dysfunction; its emergence should trigger closer surveillance with urine protein quantification, serum creatinine monitoring, and early nephrology consultation. The pattern of new‐onset or rapidly worsening hypertension coupled with proteinuria often signals evolving endothelial injury and may precede renal‐limited thrombotic microangiopathy. Clinicians should maintain a high index of suspicion for TMA in patients exhibiting nephrotic syndrome while on anti‐VEGF/TKI treatment, particularly when accompanied by signs of hemolysis or thrombocytopenia. Early diagnosis and prompt intervention, including the cessation of the offending agent, may lead to improved renal function and better overall patient prognosis. Routine incorporation of standardized blood pressure logs and spot protein‐to‐creatinine ratio testing into oncology follow‐up can allow for earlier dose adjustments or therapeutic pauses, potentially averting irreversible renal damage. Multidisciplinary coordination between oncology and nephrology is therefore essential for the safe continuation of VEGF/TKI‐based therapy and optimization of both renal and oncologic outcomes. As the use of immunotherapy continues to expand, further research is needed to elucidate the mechanisms of drug‐induced TMA and to establish effective monitoring and management strategies, ultimately enhancing patient safety and treatment efficacy in those undergoing treatment for mRCC and other malignancies.

## Author Contributions

**Sundus Sardar:** conceptualization, writing – original draft, writing – review and editing. **Ahmad Matarneh:** writing – review and editing. **Omar K. Salameh:** writing – review and editing. **Abdel‐Rauof M. Akkari:** writing – review and editing. **Monika Joshi:** writing – review and editing. **Erik Washburn:** writing – review and editing. **Nasrollah Ghahramani:** conceptualization, supervision, writing – review and editing. **Naman Trivedi:** conceptualization, supervision, writing – review and editing.

## Funding

The authors have nothing to report.

## Ethics Statement

The authors have nothing to report.

## Consent

Written informed consent was obtained from the patient to publish this case report.

## Conflicts of Interest

The authors declare no conflicts of interest.

## Data Availability

Data sharing not applicable to this article as no datasets were generated or analyzed during the current study.
